# In preprints: the problem of producing precise patterns

**DOI:** 10.1242/dev.200710

**Published:** 2022-03-30

**Authors:** Sally Lowell, Guillaume Blin

**Affiliations:** Centre for Regenerative Medicine, Institute for Stem Cell Research, School of Biological Sciences, University of Edinburgh, 5 Little France Drive, Edinburgh EH16 4UU, UK

Much of the beauty and mystery of development comes down to the question of how embryos set up patterns of gene expression. Four recent preprints tackle this fascinating question by combining quantitative imaging, modelling, experimental embryology and careful conceptual thinking.

One particularly beautiful example of patterning occurs during somitogenesis. During this process, regular stripes in gene expression emerge sequentially along the pre-somitic mesoderm (PSM), prefiguring the formation of ribs and associated muscles. This event is governed by two factors: a cell-autonomous ‘clock’ (i.e. repeated ‘tick-tock’ oscillations of gene expression) and a coordinated ‘wavefront’ (i.e. a differentiation event that occurs at defined anterior-posterior locations; Gomez et al., 2008; Palmeirim et al., 1997).

The position of the differentiation wavefront has been proposed to be dictated entirely by extrinsic signals, yet when Rohde and colleagues (Rohde et al., 2021 preprint) isolated individual zebrafish PSM cells in the absence of exogenous growth factors, they found that these cells exhibit a trajectory of transient oscillatory clock dynamics and differentiation similar to that seen in intact embryos, albeit with a higher variance. This surprising result, which is based on experimental data from live reporters and interpreted using mathematical modelling, indicates that PSM cells possess not only an intrinsic oscillator but also an intrinsic timer that is started upon exit of a cell from the tailbud and ultimately tells cells when to differentiate. This noisy timer can be influenced by extrinsic cues but is not dependent on them. The molecular basis for this intrinsic timer is not yet known and remains an area ripe for future investigation.

The story continues with a pair of interconnected preprints (Fulton et al., 2022 preprint; Spiess et al., 2022 preprint) that address the intriguing question of how a stable pattern, such as that associated with the spatially defined ‘wavefront’ of differentiation in the PSM, can emerge amid highly dynamic cell movements.

Fulton et al. first performed experiments broadly similar to those carried out by Rohde et al. to confirm that PSM cells possess a cell-intrinsic differentiation timer. They then labelled coherent groups of cells in the progenitor zone to investigate how cells move over time. After 3 h, some particularly speedy cells had already raced away into the differentiation zone, whereas others had barely moved from their starting point. So, to achieve coherent patterning of cell fate, it seems that the far-ranging cells must somehow accelerate their intrinsic differentiation timer by just the right amount, whereas more sluggish cells must down-tune their own timer to a correspondingly sluggish rate.

In general, the timing of differentiation is governed by gene regulatory networks (GRNs). In some systems, such as the fly blastoderm (Crombach et al., 2016; Verd et al., 2014) and the vertebrate neural tube (Kicheva et al., 2014; Sagner and Briscoe, 2017), the logic of GRNs has been deduced based on measurements of signalling activity and transcription factor expression over time. Cell movements in these tissues are so slow that they can be ignored, but that is not the case in the PSM (Thomson et al., 2021). To infer GRNs in the PSM, Spiess et al. therefore needed to measure the expression of multiple transcription factors and signalling pathways in cells as they are moving around in live tissues, an endeavor hampered by the limited number of live reporters that can be imaged simultaneously. They overcame this problem by measuring a broader panel of markers in ‘snapshot’ images and then superimposing these measurements onto individual cell trajectories, obtained by tracking cells within an *in toto*-imaged embryo, in order to approximate dynamic changes in gene expression.

From these data, they inferred a range of possible GRNs, which they could then test using ‘live modelling’, i.e. by simulating the effects of candidate GRNs in individual cells over time. This enabled the authors to identify a simple GRN that could account for the experimental observations (not only from embryos but also from isolated PSM cells) and that was consistent with information from the literature. They propose that, because cells move relative to defined signalling centres, cell movements dictate the strength and duration of signalling exposure – information that is then interpreted by the intracellular GRN in just the right way to generate a coherent pattern. Impressively, this GRN went beyond explaining the broad regionalisation of cell identities. It also predicted low-level ‘aberrant’ heterogeneity of cell fates within the progenitor zone, a prediction that was subsequently confirmed using sensitive detection methods.

In summary, this new methodology reveals a simple GRN that explains how stable patterns of gene expression can emerge in the context of extensive cell movements. Given that cells tend to move around a lot during many other stages of embryonic development, and probably also during the formation of organoids (Huch et al., 2017) and in other *ex vivo* models of development (Hashmi et al., 2021 preprint), this approach will be broadly applicable. Furthermore, because the method generates a range of plausible alternative GRNs, it could be used to explore how GRNs evolve over evolutionary time to adapt the body plan to novel environments.

Some questions remain. For example, the model of Fulton et al. explains patterning based on cell movements, long-range signalling and an intrinsic timer, but could there be an additional influence from local cell-cell communication?

Another recent preprint (Lee et al., 2022 preprint) tells us that cells do indeed talk to their neighbours to refine patterning at least in some contexts. In this study, Lee et al. focussed on the formation of the primitive streak at gastrulation. This process is governed by gradients of long-range signals, similar to those that influence differentiation in the PSM. Do cells interpret their position in these gradients autonomously? This may be possible, but an alternative is a ‘neighbourhood watch’ model, in which cells sense and respond to signalling differences from their neighbours. Using mathematical modelling, Lee et al. identified particular manipulations of exogenous signals that should produce different consequences depending on which model is correct. These manipulations, performed in the experimentally tractable chick model, confirmed that the neighbourhood watch model best fits the experimental observations.

Taken together, these four preprints exemplify the power of combining quantitative imaging and modelling in the context of model systems that are readily amenable to manipulation. Moreover, they reveal the internal logic of programmes that integrate dynamically changing sources of information to generate the body plan.

It is particularly intriguing that these programmes produce certain imperfections in the coherence of patterning (Fulton et al., 2022 preprint). This leaves us with one final question: is this heterogeneity entirely ‘aberrant’ or could it be helpful in some way? David Bowie once said, ‘I thrive on mistakes’. Is it possible that the embryo does too?

## References

[R1] Crombach A, Wotton KR, Jiménez-Guri E, Jaeger J (2016). Gap gene regulatory dynamics evolve along a genotype network. Mol Biol Evol.

[R2] Fulton T, Spiess K, Thomson L, Wang Y, Clark B, Hwang S, Paige B, Verd B, Steventon B (2022). Cell rearrangement generates pattern emergence as a function of temporal morphogen exposure. bioRxiv.

[R3] Gomez C, Özbudak EM, Wunderlich J, Baumann D, Lewis J, Pourquié O (2008). Control of segment number in vertebrate embryos. Nature.

[R4] Hashmi A, Tlili S, Perrin P, Martinez-Arias A, Lenne P-F (2021). Cell-state transitions and collective cell movement generate an endoderm-like region in gastruloids. bioRxiv.

[R5] Huch M, Knoblich JA, Lutolf MP, Martinez-Arias A (2017). The hope and the hype of organoid research. Development.

[R6] Kicheva A, Bollenbach T, Ribeiro A, Valle HP, Lovell-Badge R, Episkopou V, Briscoe J (2014). Coordination of progenitor specification and growth in mouse and chick spinal cord. Science.

[R7] Lee HC, Hastings C, de Oliveira NMM, Pérez-Carrasco R, Page KM, Wolpert L, Stern CD (2022). “Neighborhood watch” model: embryonic epiblast cells assess positional information in relation to their neighbors. bioRxiv.

[R8] Palmeirim I, Henrique D, Ish-Horowicz D, Pourquié O (1997). Avian hairy gene expression identifies a molecular clock linked to vertebrate segmentation and somitogenesis. Cell.

[R9] Rohde LA, Bercowsky-Rama A, Negrete J, Valentin G, Naganathan SR, Desai RA, Strnad P, Soroldoni D, Jülicher F, Oates AC (2021). Cell-autonomous generation of the wave pattern within the vertebrate segmentation clock. bioRxiv.

[R10] Sagner A, Briscoe J (2017). Morphogen interpretation: concentration, time, competence, and signaling dynamics. Wiley Interdiscipl Rev Dev Biol.

[R11] Spiess K, Fulton T, Hwang S, Toh K, Saunders D, Paige B, Steventon B, Verd B (2022). Approximated Gene Expression Trajectories (AGETs) for gene regulatory network inference on cell tracks. bioRxiv.

[R12] Thomson L, Muresan L, Steventon B (2021). The zebrafish presomitic mesoderm elongates through compaction-extension. Cells Dev.

[R13] Verd B, Crombach A, Jaeger J (2014). Classification of transient behaviours in a time-dependent toggle switch model. BMC Syst Biol.

